# Task Profiles of Academically Qualified Psychiatric Nurses in Germany: Results of a Cluster Analysis

**DOI:** 10.1111/jpm.13153

**Published:** 2025-02-07

**Authors:** Stefan Scheydt, André Nienaber, Martin Holzke

**Affiliations:** ^1^ Central Institute of Mental Health Mannheim Germany; ^2^ German Center for Mental Health (DZPG), Partner Site Mannheim‐Heidelberg‐Ulm Mannheim Germany; ^3^ Bern University of Applied Sciences Department of Health Bern Switzerland; ^4^ University Psychiatric Clinics Basel Basel Switzerland; ^5^ Center for Psychiatry South Wuerttemberg Ravensburg‐Weissenau Germany; ^6^ University Ulm Ulm Germany

**Keywords:** advanced practice nursing, cluster analysis, cross‐sectional study, Germany, mental health nursing, nurses' roles, psychiatry

## Abstract

**Introduction:**

As little clarity exists regarding the roles of academically qualified nurses in Germany it is not certain that nurses who call themselves “nursing experts” actually perform the tasks of a nursing expert or APN. An important aspect of the present “Study on the situation of academically qualified nurses in psychiatric care contexts in Germany” (AkaPP study) was therefore to identify profiles or clusters based on the tasks and activities performed by academically qualified psychiatric nurses in Germany.

**Aim:**

To identify possible clusters of academically qualified psychiatric nurses working in direct care practice, nursing development or nursing research positions in relation to their self‐described tasks and activities.

**Method:**

Data were collected via an online survey between August and November 2020. The target group of the study was academically qualified nurses in Germany working in a psychiatric‐psychosocial healthcare institution. The group of interest for the analysis was the subgroup of nurses in a “direct care and scientific nursing role” (academically qualified nurses working in direct patient care, nursing development or nursing research positions; *n* = 105 valid cases). A hierarchical cluster analysis was carried out using the Ward method on the basis of the tasks and activities described by the participants in the questionnaire. The identified clusters were described in terms of descriptive statistics against the background of previously defined content characteristics and compared for noticeable differences. Reporting was performed according to the STROBE checklist.

**Results:**

Cluster analysis revealed the following seven clusters of academically prepared nurses: (1) Practice Development and Consultative Expertise, (2) Basic Nursing Practice, (3) Advanced Practice Development and Nursing Research, (4) Specialised and Expanded Psychiatric Nursing Practice, (5) Basic Psychiatric Nursing Practice, (6) Direct Patient Care and Basic Practice Development and (7) Advanced Psychiatric Nursing Practice.

**Discussion and Implications for Practice:**

The clusters identified provide a nuanced understanding of the roles of graduate psychiatric nurses in Germany. This insight helps to tailor staffing structures and training programs to the needs of psychiatric care settings. By delineating distinct role profiles, healthcare institutions and policymakers can optimise resource allocation, enhance interdisciplinary collaboration and ultimately improve patient outcomes. This research provides guidance for refining clinical practice models and promoting professional development in psychiatric nursing contexts.

**Summary**

- What is known on the subject?
  ○ Germany lags far behind international standards in nursing education and training. This is particularly evident in higher education and in the deployment of academically qualified nurses in nursing practice.
  ○ There is no clarity about the roles of academically qualified nurses in Germany. It is not certain that nurses who call themselves “nursing experts” actually perform the tasks of a nursing expert or APN.
- What the paper adds to existing knowledge?
  ○ Statistical methods were used to develop role profiles based on the actual tasks and activities performed by academically qualified psychiatric nurses.
  ○ An empirically based model of the role profiles of academically trained nurses was developed, which complements existing competency models and classifies nursing roles into basic and advanced roles.
- What are the implications for practice?
  ○ The results provide a solid basis for a better understanding and clear definition of the role of academic nurses in psychiatric setting of the German‐speaking countries.

## 1 Background

Mental health care is facing major challenges that are associated with increasing complexity in service delivery. Influencing factors include, for example, changes in future patient characteristics (e.g., ageing population, migrants with traumatic experiences) and the corresponding care requirements with simultaneous changes in mental healthcare structures (e.g., urban–rural gap, shortage of physicians and therapists, increasing digitalisation of services) (Giacco et al. 2016; Priebe et al. 2019; Thornicroft et al. 2016). These challenges mean that psychiatric nursing in future care models will have to act increasingly autonomously and take on correspondingly expanded or advanced and highly specialised tasks (Scheydt 2024; Wand et al. 2022). To provide such extended or advanced and highly specialised care, nurses need to be trained to the appropriate level (Daly and Carnwell 2003; Scheydt and Holzke 2018b).

However, as recent studies have shown (e.g., Lehmann et al. 2019), Germany lags far behind international standards in terms of (university‐based) nursing education and training. This is particularly evident in higher education and in the deployment of academically qualified nurses in nursing practice. While other countries such as the Netherlands (45%), Canada (61%), the UK and Sweden (both 100%) have a very high proportion of nurses with a university degree, the proportion of nurses with a university degree in Germany is only around one to 2% (Lehmann et al. 2019). This is understandable, as nurses in Germany have traditionally been trained in vocational schools rather than universities, as is the case in many other countries. In most countries in Europe and beyond, the nursing profession requires a university degree (Lehmann et al. 2019). In Germany, there are various options for further education, both vocational and higher education. However, higher education for nurses in Germany is still in its infancy. There are many reasons for this, most of which lie in the historical development of the nursing profession in Germany. These developments would fill a separate article, but are briefly described in the box below (see Box 1).

BOX 1 **Why is the academisation of nursing in Germany lagging behind international developments? An attempt at a brief summary of possible (historical) reasons.**

Although the first university nursing programme was established in Germany as early as 1913, this development was halted by the Nazi regime and the Second World War. It would be more than 40 years before another attempt was made to academise nursing in Germany. It was not until the 1990s that the first degree programmes in nursing management and nursing education were offered (Heitmann and Reuter 2019; Schulz and Sauter 2015). The first bachelor's degree in nursing with a focus on nursing practice was introduced in 2003. Master's programmes followed in 2006, and the first doctoral opportunities did not appear until the 2010s. There are several reasons for this late development of the academisation of nursing in Germany. One main reason is certainly the traditional and rather backward‐looking image of nursing after the Nazi era and the Second World War (Bischoff‐Wanner 2011; Neumann 2009; Steppe 2000). Other reasons lie in the established and funded dual education system and the perception of nursing as a practice‐oriented profession, as well as in the structural characteristics of the German nursing system. In addition, there is no national register for nursing professions. Although there are individual professional associations, the degree of organisation among nurses in Germany is very low.

The fact that the academisation of nursing in Germany is lagging behind international standards is also reflected in the research on these issues. In the international context, the aspects of academisation of nursing and the integration of academically qualified nurses have been the subject of much research for decades. Several publications have described cross‐sectional studies examining the situation of academically qualified nurses, mostly in the role of advanced practice nurses. Some publications focus on the psychiatric care setting (e.g., Allen 1998; Campbell et al. 1998; Chien and Ip 2001; Delaney et al. 2019; Drew and Delaney 2009; Jones 2018; Sharrock et al. 2008). More recent findings based on empirical data on the identification of specific nursing practice roles and practice profiles are provided by Gardner et al. (2016), who used cluster analysis to identify the practice patterns of Australian nurses by job description. However, these findings do not address the roles of psychiatric nurses. Two reviews by Scheydt and Hegedüs (2021) and Hurley et al. (2022) described the clinical roles of psychiatric nurses, but at the level of tasks and activities rather than differentiating professional role profiles between basic and advanced nursing.

In German‐speaking countries, too, topics related to the academisation of nursing have attracted attention in both research and nursing practice. This is reflected, for example, in the increasing number of studies and corresponding publications in recent years. While the aspects of role development and role implementation or the views on the tasks and role understanding of academically qualified nurses are primarily examined (Baumgartner et al. 2023; von Dach et al. 2023; Doppelfeld et al. 2023; Laimbacher et al. 2023; Möcking and Hosters 2023; Scheydt and Holzke 2018b; Scheydt et al. 2020; Schlunegger et al. 2023; Schönbächler Marcar and Keller 2022; Seismann‐Petersen et al. 2023; Weidling et al. 2023; Zúñiga et al. 2022), only a few cross‐sectional studies could be identified that examined the situation of academically qualified nurses (Bergjan et al. 2021; Mertens et al. 2019; Tannen et al. 2017; Wissenschaftsrat 2022) and only a miniscule proportion of the identified studies are from the psychiatric field (Laimbacher et al. 2023; Scheydt and Holzke 2018b; Scheydt et al. 2020; Weidling et al. 2023). The studies identified so far deal with the topics of role development, role implementation and role understanding mainly anecdotally or in the context of (individual) case studies. There is no systematic cross‐sectional study in the German‐speaking area that provides valid statements based on a larger sample.

Against this background, the Research Group “Mental Health Nursing” (Central Institute of Mental Health, Mannheim, Germany), together with colleagues from the Center for Psychiatry South Wuerttemberg, has conducted the “Study on the situation of academically qualified nurses in psychiatric care contexts” (AkaPP study) (Scheydt et al. 2021). The overall aim of this study was to generate extended data on the use of academically qualified nurses, particularly in psychiatric‐psychosocial practice areas in Germany. In particular the roles performed, concrete areas of deployment, facilitators and barriers to deployment in direct patient care, as well as other selected structural data and framework conditions were collected in order to explore the status of the academisation of psychiatric nursing. Since there are no legal stipulations or regulations in Germany regarding the specific role profiles, an important aspect of the study was the explorative analysis of the roles of academically qualified psychiatric nurses.

## 2 Objectives

The aim of this study was to identify possible clusters of academically qualified nurses in terms of their tasks and activities. Against this background, the following research questions guided our work: (a) Which clusters can be identified in the subgroup of academic nurses working in direct care practice, practice development or nursing research positions based on the tasks and activities performed? (b) What are the underlying characteristics of these clusters?

## 3 Methods

### 3.1 Study Design and Background

As part of the AkaPP study (Scheydt et al. 2021), a hierarchical cluster analysis using the Ward method was conducted within the subgroup “nurses in direct care and scientific nursing roles” against the background of the objectives described above. The AkaPP study was conducted as a nationwide cross‐sectional questionnaire study. The study was reported following the STROBE (Strengthening the Reporting of Observational Studies in Epidemiologic Studies) guideline for reporting (von Elm et al. 2007).

### 3.2 Data Collection and Sampling

Data collection took place between August and November 2020 via an online survey. A self‐developed questionnaire was used for the survey, based on a systematic literature review on regarding the academisation of (psychiatric) nursing. The content of the questionnaire created in SoSci‐Survey (https://www.soscisurvey.de/) was discussed and agreed upon by the authors. This made it possible to specify the questions, add missing aspects, make linguistic adjustments and optimise the order of the questions. The questionnaire was approved on this basis was then pre‐tested by external experts (two nursing scientists with many years of experience in questionnaire research and a psychiatric background) to check the validity and comprehensibility of the content (Häder 2015). The specific content of the questionnaire has been reported elsewhere (Scheydt et al. 2021).

The analytical focus of the cluster analysis was the category “Tasks and activities” (FR06, see Appendix S1), which was developed on the basis of two systematic literature reviews (Scheydt and Hegedüs 2021; Scheydt and Holzke 2018a). The different individual variables in the “Tasks and activities” category are listed in Table S1. For a clearer presentation of the results, the individual variables in the category “Tasks and activities” (FR06) have been grouped into domains (see Table S2).

### 3.3 Sample and Description of the Analysed Subgroup

The target population of the AkaPP study was academically qualified nurses in Germany working in a psychiatric‐psychosocial healthcare setting. Study participants were recruited through social networks (e.g., Facebook, Twitter, Instagram) and email distribution lists of various professional societies and specialist departments with a focus on psychiatry and mental health. A total of 185 academically trained nurses participated in the survey. As there is no obligation to register professional nurses in Germany no statement can be made about the basic population. The total sample could be divided into five subgroups based on the described function designations (FR12): (a) direct care and scientific nursing role (*n* = 109), (b) nursing management role (*n* = 31), hybrid nursing practice and management role (*n* = 16), educational role (*n* = 7) and other (*n* = 6). The group of interest for this analysis was the subgroup “direct care and scientific nursing role”, i.e., academically qualified nurses working in direct care practice, nursing development or nursing research positions. Of the 109 cases in this subgroup, 105 cases were identified as valid (test criterion: FR06 was fully completed) and were therefore included in the analysis.

### 3.4 Statistical Analysis and Selection of the Appropriate Number of Clusters

Statistical analysis was performed using hierarchical cluster analysis (Ward's method, Euclidean squared distance measure). Prior to the cluster analysis, the data set was randomly sorted several times to eliminate any bias in the results due to possible structures within the data set. In addition, the so‐called split‐half method was used to check the robustness of the cluster solution (Backhaus et al. 2023). This involved randomly dividing the analysed data into two equal halves and performing a cluster analysis on each group using the same procedure. The high level of consistency in the results suggests that the cluster solution has an adequate degree of stability. In a final step, the identified clusters were described by descriptive statistics against the background of the previously defined content characteristics and analysed comparatively for their striking differences.

Since there are no goodness‐of‐fit indices for cluster analyses, the selection of the appropriate number of clusters was determined by a combination of analysis of the scree plot (analysis of the change in the measure of heterogeneity over the course of the fusion process using the so‐called elbow test) and visual analysis of the dendrogram (visual representation of possible groupings). Finally, the expert opinion of the members of the research group, based on many years of experience in psychiatric practice and nursing research, was considered to agree on the final number of clusters (Backhaus et al. 2023; Bortz and Schuster 2010). In addition, the strength of the effect of the cluster variables used on cluster membership was calculated for the different cluster solutions (from 5 to 10 clusters) in order to obtain possible indications of the number of clusters based on the strength of the effect. The Cramer's V correlation measure was calculated for this purpose. All statistical data analysis was performed using IBM SPSS statistical software (version 27).

### 3.5 Ethics and Informed Consent

Ethical approval of the study was granted by the Ethics Committee II of the Ruprecht‐Karls‐University Heidelberg, Medical Faculty Mannheim (No. 2024–582). Participation in the online survey was voluntary and based on informed consent. The data generated by the study were processed, analysed and stored in anonymised form in accordance with generally accepted European data protection guidelines. As the lists of identifying data of the participants are stored separately from the data of the online survey, it is not possible to draw conclusions about individual persons. Personal data will be stored for a maximum of 2 years after evaluation and then destroyed in accordance with data protection guidelines.

## 4 Results

**FIGURE 1:**
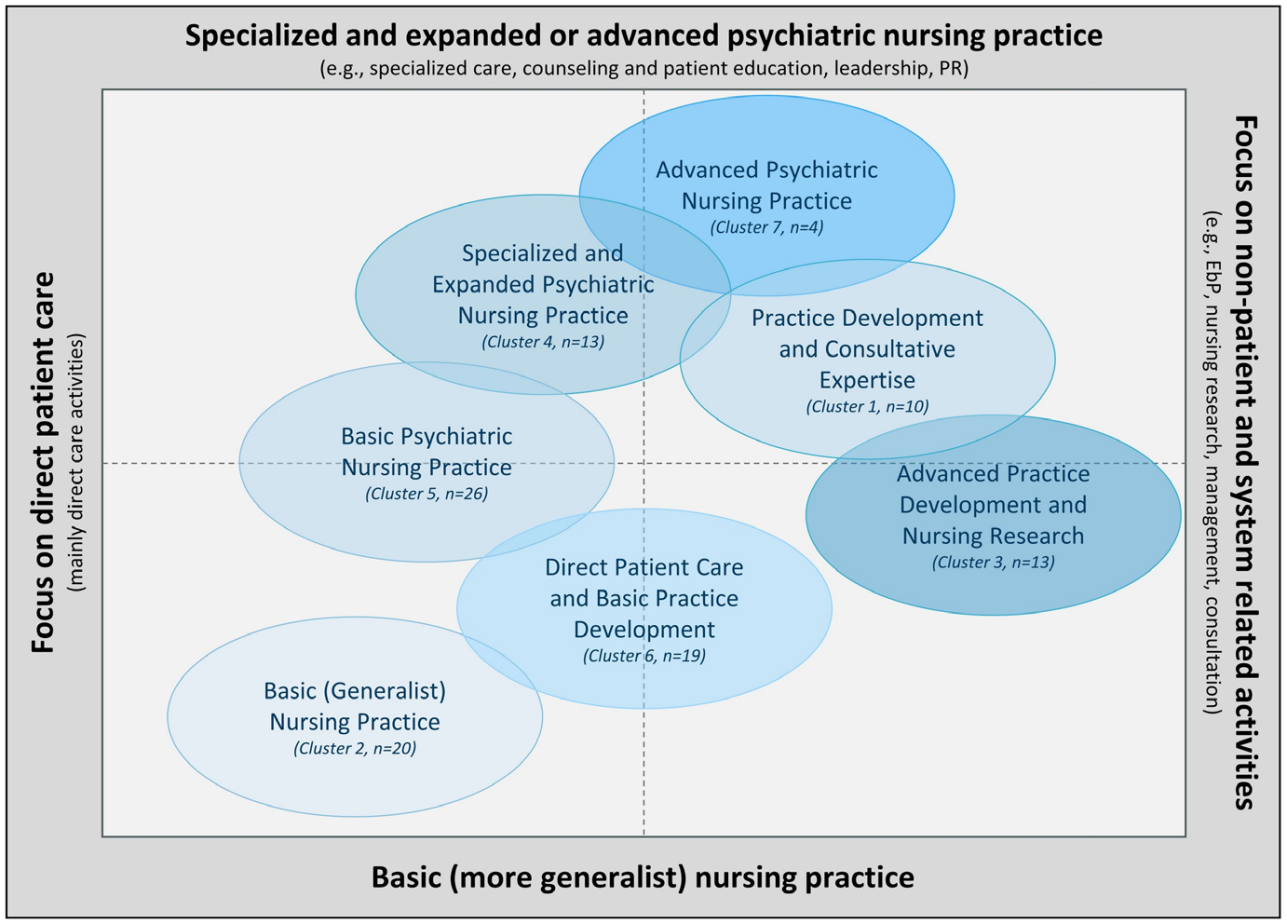
Graphical classification and delineation of clusters on the dimensions. EbP, Evidence‐based Practice; PMH, Psychiatric and Mental Health; PR, Public Relations; PR, public relations.

The cluster analysis revealed seven clusters of the tasks and activities of academically qualified nurses. The seven clusters were named as follows: (1) Practice Development and Consultative Expertise, (2) Basic Nursing Practice, (3) Advanced Practice Development and Nursing Research, (4) Specialised and Expanded Psychiatric Nursing Practice, (5) Basic Psychiatric Nursing Practice, (6) Direct Patient Care and Basic Practice Development and (7) Advanced Psychiatric Nursing Practice. The specific characteristics of each cluster are summarised in Table 1. The frequency distribution of the items within the cluster variable “Tasks and Activities” (FR06) is illustrated in Table 2. A summary graphical systematisation and delineation of the described clusters on the dimensions (a) patient proximity of activities (“Focus on direct patient care” versus “Focus on non‐patient and system related activities”) and (b) specialisation and expansion/advancement of practice (“Basic nursing practice” versus “Specialized and expanded or advanced psychiatric nursing practice”) is shown in Figure 1 below.

**TABLE 1:** Overview of cluster characteristics.

Characteristics	Cluster							Total
	1	2	3	4	5	6	7	
	*n* = 10 (9.5%)	*n* = 20 (19.0%)	*n* = 13 (12.4%)	*n* = 13 (12.4%)	*n* = 26 (24.8%)	*n* = 19 (18.1%)	*n* = 4 (3.8%)	*n* = 105 (100.0%)
**Age**								
20 to 30 years	10.0%	50.0%	15.4%	46.2%	46.2%	42.1%	0.0%	37.1%
31 to 40 years	50.0%	20.0%	38.5%	30.8%	30.8%	26.3%	25.0%	30.5%
41 to 50 years	10.0%	20.0%	30.8%	15.4%	11.5%	21.1%	50.0%	19.0%
51 to 60 years	10.0%	10.0%	15.4%	7.7%	11.5%	10.5%	25.0%	11.4%
61 years or older	20.0%	0.0%	0.0%	0.0%	0.0%	0.0%	0.0%	1.9%
**Gender**								
Female	50.0%	80.0%	61.5%>	69.2%	76.9%	73.7%	25.0%	69.5%
Male	50.0%	20.0%	38.5%	30.8%	23.1%	26.3%	75.0%	30.5%
**Qualification: professional licence and formal specialisation**								
Professional licence in nursing (“registered nurse”)	100.0%	95.0%	76.9%	91.7%	92.3%	94.7%	100.0%	91.4%
Formal specialisation in psychiatric nursing care	70.0%	42.1%	61.5%	61.5%	50.0%	42.1%	50.0%	51.4%
**Qualification: highest academic degree**								
Bachelor	70.0%	90.0%	46.2%	92.3%	76.9%	78.9%	0.0%	74.3%
Diploma	0.0%	5.0%	7.7%	0.0%	7.7%	0.0%	0.0%	3.8%
Master	30.0%	5.0%	30.8%	7.7%	15.4%	21.1%	100.0%	20.0%
Doctorate	0.0%	0.0%	15.4%	0.0%	0.0%	0.0%	0.0%	1.9%
**Functional designation**								
Registered Nurse	10.0%	90.0%	0.0%	15.4%	65.4%	26.3%	25.0%	42.3%
Specialised Nurse	0.0%	5.0%	0.0%	7.7%	19.2%	5.3%	0.0%	7.7%
Expert Nurse/Advanced Practice Nurse	20.0%	0.0%	23.1%	76.9%	3.8%	31.6%	50.0%	23.1%
Practice Development Nurse	70.0%	0.0%	46.2%	0.0%	0.0%	10.5%	25.0%	15.4%
Nursing Scientist	0.0%	0.0%	30.8%	0.0%	0.0%	5.3%	0.0%	4.8%
Quality Management Nurse	0.0%	0.0%	0.0%	0.0%	3.8%	21.1%	0.0%	4.8%
Other	0.0%	5.0%	0.0%	0.0%	3.8%	0.0%	0.0%	1.9%
**Patient closeness of the activity**								
Clinically oriented: close to the patient	50.0%	100.0%	0.0%	100.0%	100.0%	68.4%	75.0%	76.9%
Non‐clinically oriented: activity away from the patient	50.0%	0.0%	100.0%	0.0%	0.0%	31.6%	25.0%	23.1%
**Change of role and tasks after graduation**								
Applies (yes)	70.0%	10.5%	84.6%	92.3%	42.3%	84.2%	100.0%	60.0%
**Research activity 36 months before survey**								
Applies (yes)	60.0%	15.0%	100.0%	38.5%	30.8%	52.6%	100.0%	46.7%
**Professional experience**								
… in professional nursing care per se	20.3 years	9.5 years	17.2 years	11.6 years	11.0 years	12.6 years	23.2 years	13.2 years
… in the field of psychiatric nursing/psychiatry	15.0 years	10.2 years	15.5 years	9.9 years	9.6 years	9.2 years	19.0 years	11.3 years
… since last university degree	3.6 years	3.6 years	3.6 years	2.9 years	4.4 years	3.1 years	3.7 years	3.6 years

**TABLE 2:** Frequency distribution and significance level of the items within the cluster variable “tasks and activities” (FR06).

Characteristics	Cluster							Total
	1	2	3	4	5	6	7	
	10 (9.5%)	20 (19.0%)	13 (12.4%)	13 (12.4%)	26 (24.8%)	19 (18.1%)	4 (3.8%)	105 (100.0%)
**Tasks and activities (FR06): Main variables of the cluster analysis**								
Basic close‐to‐patient nursing care (analogous to vocational training)	20.0%	100.0%	0.0%	92.3%	80.8%	63.2%	75.0%	66.7%
Specialised and expanded/advanced close‐to‐patient care	30.0%	0.0%	0.0%	92.3%	46.2%	5.3%	100.0%	30.5%
Expanded medical‐diagnostic tasks (e.g., assessment, screening, …)	20.0%	0.0%	0.0%	23.1%	30.8%	10.5%	75.0%	17.1%
Specific tasks in health promotion and disease prevention	10.0%	0.0%	0.0%	53.8%	26.9%	5.3%	100.0%	19.0%
Patient and family counselling	50.0%	0.0%	30.8%	53.8%	65.4%	26.3%	75.0%	39.0%
Patient education	10.0%	0.0%	7.7%	76.9%	53.8%	21.1%	100.0%	32.4%
Performing nursing consultations	30.0%	0.0%	0.0%	30.8%	3.8%	0.0%	0.0%	7.6%
Case management at the micro level (e.g., primary nursing)	10.0%	0.0%	0.0%	76.9%	46.2%	15.8%	75.0%	27.6%
Independent professional responsibility for the treatment process	10.0%	0.0%	0.0%	53.8%	34.6%	15.8%	75.0%	21.9%
Care coordination/case management (including interface management)	20.0%	0.0%	7.7%	15.4%	15.4%	5.3%	50.0%	11.4%
Leadership and management tasks (formal management)	10.0%	0.0%	7.7%	15.4%	0.0%	10.5%	75.0%	8.6%
Leadership and management tasks (professional leadership)	50.0%	0.0%	30.8%	38,5%	3.8%	42.1%	25.0%	22.9%
Development, implementation and evaluation of new concepts	100.0%	0.0%	92.3%	92.3%	34.6%	84.2%	100.0%	60.0%
Consultative expert activity	90.0%	0.0%	30.8%	76.9%	0.0%	10.5%	25.0%	24.8%
Participation in practical training (e.g., practical guidance)	20.0%	5.0%	7.7%	53.8%	11,5%	36.8%	0.0%	20.0%
Independent planning and implementation of research projects	0.0%	0.0%	53.8%	0.0%	3.8%	0.0%	50.0%	9.5%
Independent planning and implementation of PD/QD projects	100.0%	0.0%	84.6%	15.4%	0.0%	36.8%	75.0%	31.4%
Participation/collaboration in research projects	0.0%	0.0%	61.5%	0.0%	11.5%	5.3%	75.0%	14.3%
Participation/collaboration in practice/quality development	80.0%	0.0%	69.2%	38.5%	7.7%	42.1%	75.0%	33.3%
Scientific literature research to support professional decision‐making	90.0%	0.0%	92.3%	46.2%	0.0%	57.9%	100.0%	40.0%
Participation in further education and training	80.0%	0.0%	69.2%	61.5%	7.7%	36.8%	75.0%	35.2%
Teaching (e.g., in specialist training or as a lecturer at a university)	40.0%	0.0%	46.2%	15.4%	3.8%	15.8%	75.0%	20.0%
Public relations/anti‐stigma work	30.0%	0.0%	38.5%	7.7%	0.0%	0.0%	100.0%	12.4%
Networking outside the institution	90.0%	5.0%	92.3%	61.5%	11.5%	21.1%	100.0%	39.0%
Networking within the institution	40.0%	0,0%	92.3%	61.5%	7.7%	10.5%	50.0%	29.5%
Dissemination of nursing knowledge (e.g., specialist articles or lectures)	10.0%	0.0%	84.6%	23.1%	11.5%	10.5%	75.0%	21.9%

*Note:* Colour Scale and consensus definition: > 50% to 74.9% = 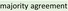; 75.0% to 89.9% = 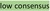; 90% or more = 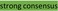.

Abbreviations: PD, practice development; QD, quality development.

### 4.1 Cluster 1: Practice Development and Consultative Expertise

Cluster 1 consists of a total of 10 academically qualified nurses, which represents a relative frequency of 9.5% within the analysed subgroup. In this cluster, 70.0% (*n* = 7) of the nurses have a bachelor's degree and 30.0% (*n* = 3) have a master's degree. All nurses in this cluster have a professional nursing licence and 70.0% (*n* = 7) have a formal specialisation in psychiatry or psychiatric nursing. The nurses in this cluster have on average 20.3 years of work experience in nursing overall (SD = 12.104 years), 15 years of professional experience in psychiatric nursing (SD = 10.759 years) and 3.6 years of professional experience since their last university degree (SD = 2.722 years). Nurses in this cluster identified their role as “Practice Development Nurse” (70.0%, *n* = 7), “Expert Nurse/Advanced Practice Nurse” (20.0%, *n* = 2) and “Registered Nurse” (10.0%, *n* = 1). Half of the nurses in this cluster do not work in direct patient care (*n* = 5). Overall, 70.0% (*n* = 7) of nurses in this cluster reported that their area of responsibility had changed since graduation.

As can be seen in Table 2, cluster 1 consists of academically qualified and professionally experienced nurses who reported their main areas of responsibility as “Development, implementation and evaluation of new concepts” (100.0%) and “Independent planning and implementation of nursing practice and quality development projects” (100.0%). Other frequently mentioned tasks in this cluster are “Scientific literature research to support professional decision‐making” (90.0%), “Consultative Expert activity on specialist topics and complex care situations/with a focus on professional and collegial advice” (90.0%), and “Networking outside the institution” (80.0%) and “Participation in further education and training” (80.0%).

### 4.2 Cluster 2: Basic (Generalist) Nursing Practice

Cluster 2 comprises 20 academically qualified nurses, which corresponds to a relative frequency of 19.0% within the analysed subgroup. Most of the nurses in cluster 2 have a bachelor's degree (90.0%, *n* = 18). In this cluster, 95.0% (*n* = 19) of the nurses have a professional nursing licence and 42.1% (*n* = 8) have an additional formal specialisation in psychiatry or psychiatric nursing. The nurses in cluster 2 have on average 9.6 years of professional experience in nursing overall (SD = 7.99 years), 10.25 years of professional experience in psychiatric nursing (SD = 8.065 years) and 3.6 years of professional experience since their last university degree (SD = 1.97 years). The nurses in cluster 2 work completely patient‐oriented in direct care practice (100.0%). Their professional title is “registered nurse” (90.0%). 18 nurses in cluster 2 (90.0%) stated that their area of responsibility had not changed since graduation. Most nurses in cluster 2 (85%, *n* = 17) reported that they had not been involved in a research project in the 36 months prior to the survey. “Basic close‐to‐patient nursing care”, analogous to vocational training, was reported as the main task in this cluster (100.0%).

### 4.3 Cluster 3: Advanced Practice Development and Nursing Research

Cluster 3 consists of 13 academically qualified nurses, representing a relative frequency of 12.4% of the analysed subgroup. In this cluster, 53.8% of the nurses have a bachelor's degree (*n* = 6) or diploma (*n* = 1), 30.8% (*n* = 4) a master's degree and 15.4% (*n* = 2) a doctorate. Ten nurses in this cluster (76.9%) have a professional nursing licence and *n* = 8 (61.5%) have an additional formal specialisation in psychiatry or psychiatric nursing. Nurses in cluster 3 have on average 17.2 years of professional experience in nursing overall (SD = 9.985 years), 15.5 years of professional experience in psychiatric nursing (SD = 7.763 years) and 3.6 years of professional experience since their last university degree (SD = 2.730 years). Nurses in cluster 3 work mainly in roles that do not involve direct patient care (100.0%) in research and development. Regarding their role function, the nurses use the following titles: “Expert Nurse/Advanced Practice Nurse” (23.1%, *n* = 3), “Practice Development Nurse” (46.2%, *n* = 6) or “Nursing Scientist” (30.8%, *n* = 4). Most nurses in cluster 3 (84.6%, *n* = 11) reported that their area of responsibility had changed since graduation. All nurses in cluster 3 reported that they had been involved in a research project within the last 36 months prior to the start of the survey.

As can be seen in Table 2, cluster 3 consists of academically qualified nurses whose main tasks are in the following areas: “Participation in or independent planning and implementation of nursing practice and quality development projects” (92.3%), “Development, implementation and evaluation of new concepts” (92.3%), “Scientific research activities to support professional decision‐making” (92.3%) and “Dissemination of nursing knowledge” (84.6%). Cluster 3 therefore has a high proportion of scientific activities in addition to the focus on practice and quality development.

### 4.4 Cluster 4: Specialised and Expanded Psychiatric/Mental Health Nursing Practice

Cluster 4 consists of 13 academically qualified nurses giving rise to a relative frequency of 12.4% of the analysed subgroup. With 92.3% (*n* = 12), the majority of the cluster has a bachelor's degree; one person (7.7%) states that they have a master's degree. In this cluster, 91.7% (*n* = 11) of the nurses have a professional nursing licence and 61.5% (*n* = 8) have an additional formal specialisation in psychiatry or psychiatric nursing. The nurses in cluster 4 have on average 11.6 years of professional experience in nursing overall (SD = 7.901 years), 9.9 years of professional experience in psychiatric nursing (SD = 4.038 years) and 2.9 years of professional experience since their last university degree (SD = 1.564 years). All nurses in cluster 4 work in direct patient care. Nurses in cluster 4 report the following job titles: “Expert Nurse/Advanced Practice Nurse” (76.9%, *n* = 10), “Registered Nurse” (15.4%, *n* = 2) and “Specialist Nurse” (7.7%, *n* = 1). Most nurses in cluster 4 (92.3%, *n* = 12) reported that their area of practice changed after graduation. In addition, 38.5% (*n* = 5) reported that they had been involved in research projects in the 36 months prior to the survey.

As can be seen in Table 2, cluster 4 consists of academically qualified nurses whose main tasks are in the areas of “Basic close‐to‐patient nursing care”, analogous to vocational training (100.0%), “Specialized and expanded/advanced close‐to‐patient care” (92.3%) and “Development, implementation and evaluation of new concepts” (92.3%). Other main areas of activity are “Patient and family counselling” (76.9%), “Case management at micro level, e.g., primary nursing” (76.9%) or “Consultative expert activity on specialist topics and complex care situations” (76.9%).

### 4.5 Cluster 5: Basic Psychiatric/Mental Health Nursing Practice

Cluster 5 consists of 26 academically qualified nurses, representing just under a quarter (24.8%) of the subgroup analysed. In this cluster, 76.9% (*n* = 20) of the nurses have a bachelor's degree, 15.4% (*n* = 4) have a master's degree and two (7.7%) have a diploma. Furthermore, 92.3% (*n* = 24) of the nurses in this cluster have a professional nursing licence and 50.0% (*n* = 13) have additional formal specialisation in psychiatry or psychiatric nursing. The nurses in cluster 5 have on average 11 years of professional experience in nursing overall (SD = 9.208 years), 9.6 years of professional experience in psychiatric nursing (SD = 7.926 years) and 4.4 years of professional experience since their last university degree (SD = 4.934 years). The nurses in cluster 5 all work in a direct care practice role proximal to the patient (100.0%). Most of the nurses in cluster 5 work in the role of “Registered Nurse” (68.0%; *n* = 17) or “Specialist Nurse” (19.2%; *n* = 5). About half (47.8%; *n* = 11) of the nurses in cluster 5 reported that their tasks had changed since graduation. In addition, 30.8% (*n* = 8) reported that they had been involved in research projects in the 36 months prior to the survey.

Cluster 5 consists of academically trained nurses whose main tasks are in the areas “Basic close‐to‐patient nursing care”, analogous to vocational training (80.8%) and “Patient education” or “Patient and family counselling” (76.9% in total).

### 4.6 Cluster 6: Direct Patient Care and Basic Practice Development

Cluster 6 consists of 19 academically qualified nurses, representing a relative frequency of 18.1% within the analysed subgroup. The majority (78.9%, *n* = 15) of nurses in cluster 6 have a bachelor's degree and 21.1% (*n* = 4) have a master's degree. 94.7% (*n* = 18) of the nurses in this cluster have a professional nursing licence and 42.1% (*n* = 8) have additional formal specialisation in psychiatry or psychiatric nursing. The nurses in cluster 6 have on average 12.6 years of professional experience in nursing (SD = 7.456 years), 9.2 years of professional experience in psychiatric nursing (SD = 7.926 years) and 3 years of professional experience since their last university degree (SD = 2.677 years). Most nurses in cluster 6 work in direct patient care (68.4%, *n* = 13), but also in “research and development” or “quality management”. Nurses in cluster 6 give the following job titles: “Registered Nurse” (25.3%, *n* = 5), “Specialist Nurse” (5.3%, *n* = 1) and “Expert Nurse/Advanced Practice Nurse” (31.6%, *n* = 6). Other job titles include “Quality Management Officer/Nurse” (21.1%, *n* = 4), “Practice Development Nurse” (10.5%, *n* = 2) and “Nursing Scientist” (5.3%, *n* = 1). Overall, 84.2% (*n* = 16) of nurses in cluster 6 reported that their tasks or roles had changed since graduation. Approximately half of the nurses in this cluster (52.6%, *n* = 10) reported that they had been involved in research projects in the 36 months prior to the survey.

Cluster 6 consists of academically qualified nurses whose main tasks are in the area of “Development, implementation and evaluation of new concepts” (84.2%), but also in the area of “Basic close‐to‐patient nursing care”, analogous to vocational training (63.2%). The other domains appear to play a rather secondary role within this cluster.

### 4.7 Cluster 7: Advanced Psychiatric/Mental Health Nursing Practice

Cluster 7 consists of 4 academically qualified nurses, representing a relative frequency of 3.8% within the analysed subgroup. All nurses in cluster 7 have a master's degree. All nurses in this cluster have a professional nursing licence and half of them have a formal specialisation in psychiatry or psychiatric nursing. Nurses in cluster 7 have on average 23.25 years of professional experience in nursing (SD = 6.602 years), 19 years of professional experience in psychiatric nursing (SD = 13.115 years) and 3.7 years of professional experience since their last university degree (SD = 3.005 years). In this cluster, 75.0% (*n* = 3) of the nurses work in direct patient care. Nurses in cluster 7 report the following job titles: “Nursing Expert/APN” (50.0%, *n* = 2), “Registered Nurse” (25.0%, *n* = 1) and “Staff Position Nursing Development” (25.0%, *n* = 1). All nurses in this cluster reported that their scope of practice had changed since graduation. All nurses in this cluster were also involved in a research project in the 36 months prior to the survey. As can be seen in Figure 1, the scope of practice of cluster 7 extends across all domains. However, there appears to be less activity in the domains “care management and systems support” and “education and teaching” than in the other domains.

## 5 Discussion

This study uses empirical data to describe the practice profile of academic psychiatric nursing or advanced psychiatric nursing practice in Germany. Cluster analysis of the tasks and activities, described by the participants, was used to identify seven clusters of academically qualified nurses. These different clusters describe different academic role profiles in psychiatric nursing, some of which differ significantly from each other in terms of practice patterns. The clusters can be grouped into two main areas: “specialized patient care” or “research and development”, on a continuum from “basic” to “specialised” to “advanced” practice (see Figure 1). This task and activity profile provides a realistic and representative picture of the academic role profiles of psychiatric nurses in Germany. It can likely be generalised to the healthcare systems in Austria and Switzerland.

The results provide a first insight into the academic landscape of psychiatric nursing in Germany. For the first time, the academisation of psychiatric nursing in Germany can be compared internationally. Similar to the study by Gardner et al. (2017), a cluster analysis is used to identify task profiles and nursing role types. In our case, however, this is done on the basis of self‐described tasks and activities and not on the basis of self‐described job titles. Similar to previous studies (e.g., Carryer et al. 2018; Duffield et al. 2021; Gardner et al. 2016; Jokiniemi et al. 2022), this study aims to practice profiles and to differentiate the different (academic) role profiles based on the underlying tasks or practice patterns.

The study results make a significant contribution to the understanding of roles in psychiatric nursing, providing an empirical approach different from most theoretical or normative prescriptions and describing roles from basic to advanced psychiatric nursing, adding to the international literature and contributing to theory building in psychiatric nursing. The clusters identified are consistent with contemporary competency models or guidelines for psychiatric/mental health nursing, such as those developed by the International Council of Nurses (2008, 2020, 2024) and Scheydt and Holzke (2018b). However, to fully capture the breadth of the field of role profiles of academically prepared nurses in mental health settings, these models need to be expanded to include research and development, management and leadership, and education and training. This can be achieved by validating the content, for example through the Delphi method.

Clear delineation of nursing roles in psychiatry promotes professional development enables optimal use and evaluation of roles at different career stages, and supports their formalisation. The prevailing approach to workforce planning focuses mainly on the number of nurses needed in relation to the number of patients, rarely considering the level of expertise and skill mix. A proper synthesis of existing knowledge can facilitate the (further) development of (existing) tools that promote appropriate role delineation and skill‐based staffing. It could be important for service providers and managers to recognise the uniqueness of each role and develop strategies for effective collaboration in order to maximise the benefits of each role.

In addition, the results can help policymakers better understand the needs and requirements of mental health care and to implement appropriate measures to promote practice, research and education. This could improve the quality of care and strengthen (mental) health care as a whole. In view of planned changes in legislation that will expand the responsibilities of qualified nursing staff (e.g., the Nursing Competence Act planned for 2025 in Germany, see German Federal Ministry of Health 2023), it is crucial to establish clear role models and competence levels. In addition, the regulatory authorised instance should regulate and monitor continuing education. It would then be necessary to be registered as a specialised or extended nurse in order to take on such (extended or advanced) nursing care tasks.

### 5.1 Limitations

Due to the specific objective and research question, only academically qualified nurses were considered in this study. However, due to the predominantly vocational training structure in Germany, they account for only about one to 2% of all professional nurses (Lehmann et al. 2019). Therefore, this study only describes the role profiles and practice patterns at the academic level, but not psychiatric nursing in general. Future studies should also include non‐academic nurses for comparison.

Participants for the study were recruited through German professional associations with a focus on psychiatric nursing. However, this may have introduced a bias due to an overrepresentation of nurses from inpatient settings. It is important to note that nurses working in outpatient and community settings were underrepresented, which may limit the interpretation of the results. It is also important to note that there is no mandatory registration for nurses in Germany. Therefore, it is not possible to draw conclusions about the total number of academically qualified nurses and the corresponding response rate.

Another limitation is the method of cluster analysis itself. Due to its exploratory nature, cluster analysis is used only to construct knowledge from the empirical data and not for inferential statistical verification. The selection of cluster solutions and interpretation of cluster content is also somewhat subjective and highly dependent on the researchers. To reduce complexity, only nurses working in direct care practice, nursing development or nursing research positions were analysed. Subsequent analysis and theory development will need to include the other nursing groups (management and education).

In addition, cluster 7 is relatively small compared to the other clusters (*n* = 4). Even if we assume that the size of cluster 7 reflects the reality of APN in Germany, the small sample size within the cluster could still lead to various limitations regarding the quality and transferability of aspects such as robustness, interpretability or susceptibility to bias. This must be taken into account when interpreting the results.

From the vantage point of the development of nursing practice, this paper does in no way solve the fundamental problem of “prescriptionism” (theoretical contemplation of the ideal roles of academically prepared mental health nurses) versus “empiricism” (actually examining the role profiles in real practice). However, this paper may serve as a impetus for the development of such a discussion.

Furthermore, no existing instrument was used to delineate advanced roles, but a specially developed item battery based on previous work on the (advanced) tasks and activities of psychiatric nursing (Scheydt and Hegedüs 2021; Scheydt et al. 2019). For future studies, an internationally established instrument such as the Advanced Practice Role Delineation Tool (e.g., Gardner et al. 2016) should be considered.

## 6 Conclusion

The AkaPP study was the first to develop an empirically derived model of the role profiles of psychiatric nurses in Germany, which complements existing competency models and classifies nursing roles between basic and advanced psychiatric nursing. Further analyses, e.g., taking into account aspects of role development, can provide in‐depth insights into the situation of academically qualified nurses in psychiatry (in Germany). Recommendations for management, practice development, education, policy development and research can be derived. For example, future‐oriented role models can be derived from the empirically generated task clusters, which can serve as a basis for further research projects on necessary competencies. These can also be used to develop curricula at the university level, such as Psychiatric/Mental Health Nurse Practitioner (PMH‐NP) and Community Mental Health Nurse (CMHN). In addition, the results can help nurses to define their role more clearly and improve their competencies to better meet the needs of patients. Nurse managers could develop a better understanding of the different role profiles and organise and structure their teams accordingly, which could lead to a more efficient division of labour and better use of resources. Identifying the different role profiles also enables healthcare professionals to better understand their roles and responsibilities and develop their professional competencies. In particular, young nurses in the early stages of their careers can benefit from the results in order to plan their individual career based on their aspirations and goals.

## 7 Implications for Practice

The clusters identified provide a nuanced understanding of the diverse roles of graduate psychiatric nurses. This comprehensive insight helps to tailor staffing structures and training programs to the specific needs of psychiatric care settings. By delineating distinct role profiles, healthcare institutions and policymakers can optimise resource allocation, enhance interdisciplinary collaboration and ultimately improve patient outcomes. This research serves as a valuable guide for refining clinical practice models and promoting professional development in psychiatric nursing contexts.

## Ethics Statement

Ethical approval of the study was granted by the Ethics Committee II of the Ruprecht‐Karls‐University Heidelberg, Medical Faculty Mannheim (No. 2024–582). Participation in the online survey was voluntary and based on informed consent. The data generated by the study were processed, analysed and stored in anonymised form in accordance with generally accepted European data protection guidelines. The lists of identifying data of the participants are stored separately from the data of the online survey. It is not possible to draw conclusions about individual persons. Personal data will be stored for a maximum of 2 years after evaluation and then destroyed in accordance with data protection guidelines. As the data protection officer of the AkaPP study, the corresponding author assumes full responsibility for the ethically correct conduct of the examination.

## Conflicts of Interest

The authors declare no conflicts of interest.

## Supporting information


Appendix S1.

## Data Availability

Research data are not shared.
