# Supplementary material for: Task Profiles of Academically Qualified Psychiatric Nurses in Germany: Results of a Cluster Analysis
Source: J Psychiatr Ment Health Nurs. 2025 Feb 7;32(3):753–65. doi: 10.1111/jpm.13153 (PMC12056471; doi:10.1111/jpm.13153)
Supplement: Supplementary file 1 — Appendix S1. [file JPM-32-753-s001.docx]

**Supporting Information**

# Online Table 1: Individual variables in the category "tasks and activities" (FR06)

| **Code** | **Variable name** | **Code** | **Variable name** |
| --- | --- | --- | --- |
| FR06_01 | Basic close-to-patient nursing care (analogous to vocational training) | FR06_14 | Consultative expert activity (e.g., specialist-collegial advice and consultation on specialist topics or complex care situations) |
| FR06_02 | Specialized and expanded/advanced close-to-patient care (e.g., psychotherapeutic interventions) | FR06_16 | Participation in practical training (e.g., practical guidance) |
| FR06_03 | Expanded medical-diagnostic tasks (e.g., assessment, screening, diagnostics) | FR06_17 | Independent planning and implementation of research projects |
| FR06_04 | Specific tasks in health promotion and disease prevention | FR06_18 | Independent planning and implementation of practice or quality development projects |
| FR06_05 | Patient and family counseling | FR06_19 | Participation/ collaboration in research projects |
| FR06_06 | Patient education | FR06_20 | Participation/ collaboration in practice/ quality development |
| FR06_07 | Performing nursing consultations | FR06_21 | Scientific literature research to support professional decision-making |
| FR06_08 | Case management at the micro level (e.g., primary nursing) | FR06_22 | Participation in further education and training |
| FR06_09 | Independent professional responsibility for the treatment process | FR06_23 | Teaching (e.g., in specialist training or as a lecturer at a university) |
| FR06_10 | Care coordination/case management (including interface management) | FR06_24 | Public relations/ anti-stigma work |
| FR06_11 | Leadership and management tasks (formal management) | FR06_25_26 | Networking outside the institution |
| FR06_12 | Leadership and management tasks (professional leadership) | FR06_27 | Networking within the institution |
| FR06_13_15 | Conceptual work/ Development, implementation, and evaluation of new concepts | FR06_28 | Dissemination of nursing knowledge (e.g., specialist articles or lectures) |

# Online Table 2: Domains of tasks and activities including the underlying single variables of the category "Tasks and activities" (FR06)

| **Domain** | | **Single variables from FR06** |
| --- | --- | --- |
| 1 | Basic close-to-patient care | Basic close-to-patient nursing care (analogous to vocational training) (FR06_01) |
| 2 | Advanced/specialized patient care and health promotion | Specialized and advanced close-to-patient care (FR06_02); Specific tasks in health promotion and disease prevention (FR06_04); Expanded medical-diagnostic tasks (FR06_03); Patient and family counseling (FR06_05); Patient education (FR06_06) |
| 3 | Care management and systems support | Case management at the micro level (FR06_08); Care coordination/case management (FR06_10); Consultation and expertise (FR06_07; FR06_14) |
| 4 | Leadership, professional networking, and public relations | Formal management and professional leadership (FR06_11; FR06_12); Public relations/ anti-stigma work (FR06_24); Networking within (FR06_27) and outside (FR06_25_26) the institution |
| 5 | Education and teaching | Participation in practical training (FR06_16); Involvement in further education and training (FR06_22); Teaching (e.g., in specialist training or as a lecturer at a university) (FR06_23) |
| 6 | Research and practice development | Independent planning and implementation of practice or quality development projects (FR06_18); Participation/ collaboration in practice/ quality development (FR06_20); Independent planning and implementation of research projects (FR06_17); Participation/ collaboration in research projects (FR06_19); Conceptual work/ Development, implementation, and evaluation of new concepts (FR06_13_15); Dissemination of nursing knowledge (FR06_28) |
